# Long-Term Prognostic Factors in Patients With Antineutrophil Cytoplasmic Antibody-Associated Vasculitis: A 15-Year Multicenter Retrospective Study

**DOI:** 10.3389/fimmu.2022.913667

**Published:** 2022-06-30

**Authors:** Qian-Qian Liao, Ya-Fei Ren, Ke-Wei Zhu, Dong Qin, Yan-Ju Mo, Shan Cong, Juan Wu, Chun-Ying Wang, Xiao-Jiao Cui, Hong-Zhen Xu, Lin-Zheng Guo, You-Yan Zhang, Hai-Xia Song, Wei Zhang, Zhe Yang, Yan-Feng Tang, Zhuo-Jun Li, Zhou-Ni Xie, Li-Mei Li, Hui-Juan Wang, Meng-Meng Zhou, Fang-Ning Wei, Peng Chen, Yu-Hong Shi

**Affiliations:** ^1^ Department of Pharmacy, People’s Hospital of Guilin, Guilin, China; ^2^ Department of Rheumatology and Immunology, Affiliated Hospital of Guilin Medical University, Guilin, China; ^3^ Department of Clinical Research, Institute of Pharmacology, Guangzhou Baiyunshan Pharmaceutical Holding Co., Ltd., Baiyunshan Pharmaceutical General Factory, Guangzhou, China; ^4^ Department of Clinical Pharmacology, Xiangya Hospital, Central South University, Changsha, China; ^5^ Department of Respiratory and Critical Care Medicine, People’s Hospital of Guilin, Guilin, China; ^6^ Department of Pharmacy, The Second Affiliated Hospital of Qiqihar Medical University, Qiqihar, China; ^7^ Department of Rheumatology, Ganzhou People’s Hospital, Ganzhou, China; ^8^ Department of Pharmacy, The Third Affiliated Hospital of Qiqihar Medical University, Qiqihar, China; ^9^ Department of Pharmacy, Personalized Drug Therapy Key Laboratory of Sichuan Province, Sichuan Academy of Medical Science and Sichuan Provincial People's Hospital, University of Electronic Science and Technology of China, Chengdu, China; ^10^ Department of Thyroid, Breast and Vascular Surgery, People’s Hospital of Guilin, Guilin, China; ^11^ Department of Rheumatology and Immunology, People’s Hospital of Guilin, Guilin, China; ^12^ Department of Pharmacy, Yangquan Coal Industry (Group) General Hospital, Yangquan, China; ^13^ Department of Pharmacy, North China Medical Health Group Xingtai General Hospital, Xingtai, China; ^14^ Department of Geriatrics, People’s Hospital of Guilin, Guilin, China; ^15^ Department of Neurology, People’s Hospital of Guilin, Guilin, China; ^16^ Department of General Medicine, People’s Hospital, Guilin, China; ^17^ School of Public Health, Guilin Medical University, Guilin, China; ^18^ School of Clinical Pharmacy, Guilin Medical University, Guilin, China

**Keywords:** ANCA-associated vasculitis (AAV), prognosis, overall survival (OS), nomogram, 5-year survival rate, decision curve analysis (DCA)

## Abstract

**Background:**

Antineutrophil cytoplasmic antibody (ANCA)-associated vasculitis (AAV) is a multisystem autoimmune disease with small-vessel involvement. In AAV, microscopic polyangiitis (MPA) and granulomatosis with polyangiitis (GPA) are major clinicopathologic variants. In addition, myeloperoxidase (MPO) and proteinase 3 (PR3) are major target antigens. The objective of the study was to explore the predictive factors for long-term survival in AAV patients.

**Materials and Methods:**

A multicenter retrospective study was carried out on 407 patients between 2005 and 2020. Clinical parameters were obtained from laboratory tests including the ANCA types, antinuclear antibody (ANA), extractable nuclear antigen (ENA), anti-streptolysin O (ASO), glomerular filtration rate (GFR), and the laboratory examinations for the blood routine, liver function, renal function, and immunity, etc. The data for clinical parameters were collected from electronic medical records (EMRs), and the data for patient survival were acquired through regular follow-up. The association of clinical parameters with overall survival (OS) along with 3-year and 5-year survival rates was analyzed, and the nomogram as a predictive model was established according to the analysis results.

**Results:**

In the present study, 336 (82.6%) patients and 46 (11.3%) patients were diagnosed with MPA and GPA, respectively. The mean and median OS for all the patients were 2,285 and 2,290 days, respectively. The 1-year, 3-year, 5-year, and 10-year cumulative survival rates for all the patients were 84.2%, 76.3%, 57.2%, and 32.4%, respectively. Univariate and multivariate survival analyses indicated that the independent prognostic factors included age, pathological categories (MPA, GPA, and other types), serum ANCA types (negative or positive for MPO and/or PR3), ANA, ASO, GFR, lymphocyte, neutrophil-to-lymphocyte ratio (NLR), and C-reactive protein (CRP), and these clinical parameters except for ASO were used to construct a nomogram. The nomogram for 3-year and 5-year survival rates had a C-index of 0.721 (95% CI 0.676–0.766). The calibration curves showed that the predicted values of the nomogram for 3-year and 5-year survival rates were generally consistent with practical observed values, and decision curve analysis (DCA) further demonstrated the practicability and accuracy of the predictive model.

**Conclusion:**

Laboratory tests at diagnosis have great significance in the prediction of long-term survival in AAV patients.

## Introduction

Antineutrophil cytoplasmic antibody (ANCA)-associated vasculitis (AAV) is a multisystem autoimmune disease characterized by inflammatory cell infiltration that mainly affects small vessels, with no or few immune deposits (1). AAV has a higher incidence in older people and is regarded as a chronic disease necessary to receive long-term immunosuppressive agents (2). The major clinicopathologic types of AAV include microscopic polyangiitis (MPA), granulomatosis with polyangiitis (GPA), and eosinophilic GPA (EGPA). Among these clinicopathologic types, MPA and GPA account for 90% of AAV cases (3).

In Europe, the prevalence of AAV has been increasing over the past two decades, due to new identification technologies and dramatic advances in therapy contributing to the improvement in AAV patient survival, and it was reported to be in the range from 46 to 184 per million (4). In addition, AAV has an incidence of 13–20 per million annually (2, 4). With respect to Asian populations, the estimated annual incidence of AAV with renal involvement was reported to be 14.8 per million according to a nationwide survey in Japan (5).

Although the introduction of immunosuppressants (e.g., cyclophosphamide, glucocorticoids, and rituximab) contributes to the improvement of the prognosis and remission rate of AAV patients, these patients remain to be at risk of various complications (2). In fact, with a continuous extension of the disease course, renal, otolaryngological, and iatrogenic damage gradually exacerbates in AAV patients. It was estimated that 1/3 of patients with AAV have more than five damaged items 7 years after AAV diagnosis (6). Thus, the risk of mortality increases as AAV progresses, leading to poor long-term prognosis in AAV patients, and it was reported that the 5-year survival rate was approximately 75% (2). However, the long-term prognosis of AAV patients has been poorly understood so far, especially in the Chinese population (7, 8).

With respect to the prognostic factors in AAV patients, several clinical factors were identified to predict overall survival (OS), such as advanced renal failure, pulmonary hemorrhage, usual interstitial pneumonia, age, Birmingham vasculitis activity score (BVAS), accompanying comorbidities, functional status, glomerular filtration rate (GFR), white blood cell (WBC), hemoglobin (Hb), complement 3 (C3), erythrocyte sedimentation rate (ESR), and serum creatinine (Scr) (9–13). Recently, it was reported that neutrophil-to-lymphocyte ratio (NLR) served as a biomarker of severe infection, vasculitis activity, relapse, renal prognosis, and all-cause mortality in AAV. However, whether NLR was related to long-term survival in AAV was controversial (14–17). Platelet-to-lymphocyte ratio (PLR) was identified to be an independent predictor of current vasculitis activity in AAV (18). Moreover, PLR was negatively correlated with GFR, and was a predictor for renal involvement (19). Liver involvement is related to the disease activity and predicts poor clinical outcomes in GPA (20). The antinuclear antibody (ANA) was associated with renal impairment as well as chronic changes in renal histopathology for AAV patients (21). In some regions, anti-streptolysin O (ASO) antibody tests are frequently requested in clinical practice, especially in rheumatology, whereas the usefulness of ASO remains elusive (22). Whether the NLR, PLR, liver involvement, ANA, or ASO had predictive value for long-term survival in AAV patients was unclear.

AAV patients underwent a series of laboratory examinations at diagnosis. In this study, the clinical parameters of AAV patients were obtained from these laboratory examinations. We investigated the association of the clinical parameters with long-term prognosis in AAV and identified the independent prognostic factors, which were used to construct a nomogram. The developed nomogram was used to predict 3-year and 5-year survival rates in AAV patients.

## Materials and Methods

### Patients

A total of 407 patients were included in the retrospective study, with a new diagnosis of AAV between August 15, 2005, and December 27, 2020, of whom 20 (4.9%), 133 (32.7%), and 254 (62.4%) were diagnosed during 2005–2010, 2011–2015, and 2016–2020, respectively. The AAV diagnosis was determined on the basis of clinical presentations combined with positive ANCA serology. All of the patients were pathologically classified as MPA, GPA, or other types in accordance with the 2012 Revised Chapel Hill criteria (1). The study was carried out at 16 centers in 9 hospitals across 7 provinces, and the leading hospital was the People’s Hospital of Guilin, Guilin, China.

Signed informed consent was unable to be obtained from every patient due to various limitations in the clinical practice. In addition, the study was a non-interventional retrospective study, without any biological tissue sample acquired from the patients. Thus, the signed informed consent was waived in the current study. The study was approved firstly by the independent Medical Ethics Committee of the People’s Hospital of Guilin (No. 2021-073KY), Guilin, China, and then was approved by the independent Medical Ethics Committee of the Affiliated Hospital of Guilin Medical University (No. 2022QTLL-05), Guilin, China. Eventually, all of the studies were approved by the local ethics committees. Verbal informed consent was obtained from each AAV patient included in our study or the family member of a dead patient during follow-up.

Exclusion criteria included age < 14 years, failure to contact, malignancy, a coexistent multisystem autoimmune condition, and HIV infection.

### Clinical Data

AAV patients underwent a series of laboratory examinations at diagnosis, including ELISA of ANCA, indirect immunofluorescence (IIF) of ANCA, blood routine, liver function, and renal function, etc. Additionally, the ANA, extractable nuclear antigen (ENA), lupus, GFR, ESR, C-reactive protein (CRP), urine protein, and rheumatoid factor (RF) were detected in most of the patients. ASO was tested in some patients.

The GFR was estimated from Scr using the classic Cockcroft–Gault formula (23). Vasculitis disease activity was evaluated using BVAS V.2 with 66 items in total (24). The PLR, NLR, and monocyte-to-lymphocyte ratio (MLR) were calculated using the counts of the platelet, lymphocyte, neutrophil, and monocyte. A/G was calculated as the ratio of albumin to globulin.

The clinical data of patients were obtained from electronic medical records (EMRs), and the prognostic information was acquired through regular follow-ups. The OS was calculated from the date of diagnosis with AAV to the date of death from any cause. For the AAV patients surviving by the end of follow-up, the date of the last follow-up was used as the censoring date. The 3-year survival rate and 5-year survival rate were calculated as the cumulative survival rates at the end of 3 and 5 years, respectively.

### Statistical Analysis

Survival curves were plotted using the Kaplan–Meier method, and the difference between/among groups was evaluated using the log-rank test. Univariate and multivariate analyses for OS were performed through Cox regression, and the hazard ratio (HR) and 95% CI were calculated using a Cox proportional hazards model. Covariates were identified based on significant prognostic values through forward stepwise regression in univariate analysis and were included in multivariate analysis. The nomogram and calibration were constructed on the basis of the results of the multivariate analysis. The 1-year, 3-year, 5-year, and 10-year survival rates were estimated using the Kaplan–Meier method. The differences between/among groups in the 3-year survival rate as well as the 5-year survival rate were evaluated using a chi-square test. Receiver operating characteristic (ROC) curves were used to evaluate the predictive value of age, BVAS, and hematological parameters for the 3-year survival rate and 5-year survival rate. ROC curves were plotted using R (version 4.1.0) analysis packages including pROC_1.18.0.1, time ROC_0.4, and survival_3.2-7. The plotting of the nomogram along with corresponding calibration curves, calculation of a C-index, and decision curve analysis (DCA) was performed using the R analysis package rms_6.2-0. The correlation between continuous variables was evaluated by Pearson’s or Spearman’s correlation coefficient in accordance with the distribution of values. Statistical analysis was performed using the IBM SPSS software version 26.0 (IBM SPSS Inc., Chicago, IL, USA), and a two-sided *p*-value of <0.05 was considered to be statistically significant.

## Results

### Clinical Characteristics at Baseline

The baseline demographic characteristics and laboratory examination results of 407 AAV patients are shown in **Table 1**. The patients with a median (25th–75th percentile) age of 60 (51–68) years at diagnosis consisted of 198 (48.6%) male patients and 209 (51.4%) female patients. A total of 336 (82.6%) patients and 46 (11.3%) patients were diagnosed with MPA and GPA, respectively. In the present study, MPA and GPA accounted for 91.4% of all AAV patients. On ANCA subtyping through ELISA analysis, 286 (70.3%), 40 (9.8%), 6 (1.5%), and 75 (18.4%) were myeloperoxidase (MPO)-ANCA, proteinase 3 (PR3)-ANCA, double positive for MPO-ANCA and PR3-ANCA, and negative for both, respectively. In the MPA patients, 275 (81.8%) patients tested positive for MPO-ANCA. In the GPA patients, 22 (47.8%) patients tested positive for PR3-ANCA (**Table S1**). For ANCA subtyping through IIF detection, 317 (77.9%), 43 (10.6%), 3 (0.7%), and 44 (10.8%) were perinuclear ANCA (p-ANCA), cytoplasmic ANCA (c-ANCA), double positive, and negative for both, respectively. The median (25th–75th percentile) Scr, GFR, ESR, and CRP were 237.2 (87.9–509.0) μmol/L, 22.93 (9.94–65.33) ml/min, 74 (44–116) mm/h, and 24.20 (7.05–84.55) mg/L, respectively. A total of 93 (22.9%), 309 (75.9%), 216 (53.1%), and 241 (59.2%) patients underwent ASO, ANA, ENA, and lupus tests, respectively. These test results showed that 39 (41.9%), 102 (33.0%), 48 (22.2%), and 105 (43.6%) were positive, respectively. There were 153 (37.6%) deaths until the end of follow-up. The mean (SE) and median (25th–75th percentile) follow-up periods for all the patients were 1,612 (80) days and 1,266 (654–2,245) days, respectively.

**Table 1:** The baseline characteristics of 407 patients with ANCA-associated vasculitis.

Characteristics	Total, n (%)	Median (25th–75th percentile)
Age at diagnosis	407 (100.0%)	60 (51–68) years
Gender		
Male	198 (48.6%)	
Female	209 (51.4%)	
BVAS		
≤10	123 (30.2%)	
>10	284 (69.8%)	
Clinicopathology		
MPA	336 (82.6%)	
GPA	46 (11.3%)	
Other types	25 (6.1%)	
ANCA ELISA only		
MPO	286 (70.3%)	
PR3	40 (9.8%)	
Double positive	6 (1.5%)	
Negative	75 (18.4%)	
ANCA IIF only		
p-ANCA	317 (77.9%)	
c-ANCA	43 (10.6%)	
Double positive	3 (0.7%)	
Negative	44 (10.8%)	
Scr	407 (100.0%)	237.2 (87.9–509.0) μmol/L
GFR	407 (100.0%)	22.93 (9.94–65.33) ml/min
ESR	387 (95.1%)	74 (44–116) mm/h
CRP	372 (92.6%)	24.20 (7.05–84.55) mg/L
WBC	407 (100.0%)	8.60 (6.52–11.80), ×10^9^/L
RBC	407 (100.0%)	2.96 (2.40–3.72), ×10^12^/L
Hb	407 (100.0%)	84 (70–106), g/L
Platelet	407 (100.0%)	255 (181–332), ×10^9^/L
Neutrophil	407 (100.0%)	6.50 (4.60–9.33), ×10^9^/L
Monocyte	407 (100.0%)	0.49 (0.34–0.71), ×10^9^/L
Lymphocyte	407 (100.0%)	1.14 (0.80–1.70), ×10^9^/L
Eosinophil	407 (100.0%)	0.10 (0.01–0.26), ×10^9^/L
Basophil	407 (100.0%)	0.02 (0.01–0.04), ×10^9^/L
PLR	407 (100.0%)	222.00 (137.83–312.96)
NLR	407 (100.0%)	5.54 (3.33–9.53)
MLR	407 (100.0%)	0.38 (0.25–0.59)
TP	407 (100.0%)	62.7 (55.9–68.4), g/L
Prealbumin	407 (100.0%)	770 (515–1,020), mg/L
Albumin	407 (100.0%)	29.9 (26.1–35.2), g/L
Globulin	407 (100.0%)	32.3 (26.5–37.6), g/L
A/G	407 (100.0%)	0.958 (0.744–1.219)
ALT	407 (100.0%)	13.3 (8.25–25.5), U/L
AST	407 (100.0%)	18.7 (14.5–29.7), U/L
TBIL	407 (100.0%)	6.4 (4.6–8.9), μmol/L
DBIL	407 (100.0%)	2.9 (1.9–3.9), μmol/L
IBIL	407 (100.0%)	4.6 (2.7–6.5), μmol/L
Total cholesterol	407 (100.0%)	4.30 (3.25–5.28), mmol/L
Triglyceride	407 (100.0%)	1.20 (0.86–1.63), mmol/L
BUN	407 (100.0%)	12.25 (6.01–20.7), mmol/L
UA	407 (100.0%)	383.3 (269.0–488.7), μmol/L
C3	359 (88.2%)	0.863 (0.711–1.073), g/L
C4	359 (88.2%)	0.231 (0.184–0.296), g/L
IgA	359 (88.2%)	2.26 (1.52–3.00), g/L
IgG	359 (88.2%)	13.68 (9.84–17.06), g/L
IgM	359 (88.2%)	0.96 (0.71–1.59), g/L
Urine protein		
Positive	282 (69.3%)	
Negative	108 (26.5%)	
Not detected	17 (4.2%)	
ASO		
Positive	39 (9.6%)	
Negative	54 (13.3%)	
Not detected	314 (77.1%)	
ANA		
Positive	102 (25.1%)	
Negative	207 (50.9%)	
Not detected	98 (24.1%)	
RF		
Positive	93 (22.9%)	
Negative	201 (49.4%)	
Not detected	113 (27.8%)	
ENA		
Positive	48 (11.8%)	
Negative	168 (41.3%)	
Not detected	191 (46.9%)	
Lupus		
Positive	105 (25.8%)	
Negative	136 (33.4%)	
Not detected	166 (40.8%)	
Death		
Yes	153 (37.6%)	
No	254 (62.4%)	

ANCA, antineutrophil cytoplasmic antibody; BVAS, Birmingham vasculitis activity score; MPA, microscopic polyangiitis; GPA, granulomatosis with polyangiitis; MPO, myeloperoxidase; PR3, proteinase 3; IIF, indirect immunofluorescence; c-ANCA, cytoplasm-ANCA; p-ANCA, peripheral-ANCA; Scr, serum creatinine; GFR, glomerular filtration rate; ESR, erythrocyte sedimentation rate; CRP, C-reactive protein; WBC, white blood cell; RBC, red blood cellt; Hb, hemoglobin; PLR, platelet-to-lymphocyte ratio; NLR, neutrophil-to-lymphocyte ratio; MLR, monocyte-to-lymphocyte ratio; TP, total protein; A/G, albumin-to-globulin ratio; ALT, alanine aminotransferase; AST, aspartate aminotransferase; TBIL, total bilirubin; DBIL, direct bilirubin; IBIL, indirect bilirubin; BUN, blood urea nitrogen; UA, uric acid; C3, complement 3; C4, complement 4; IgA, immunoglobulin A; IgG, immunoglobulin G; IgM, immunoglobulin M; ASO, anti-streptolysin O; ANA, antinuclear antibody; RF, rheumatoid factor; ENA, extractable nuclear antigen.

### Overall Survival

**Figure 1:**
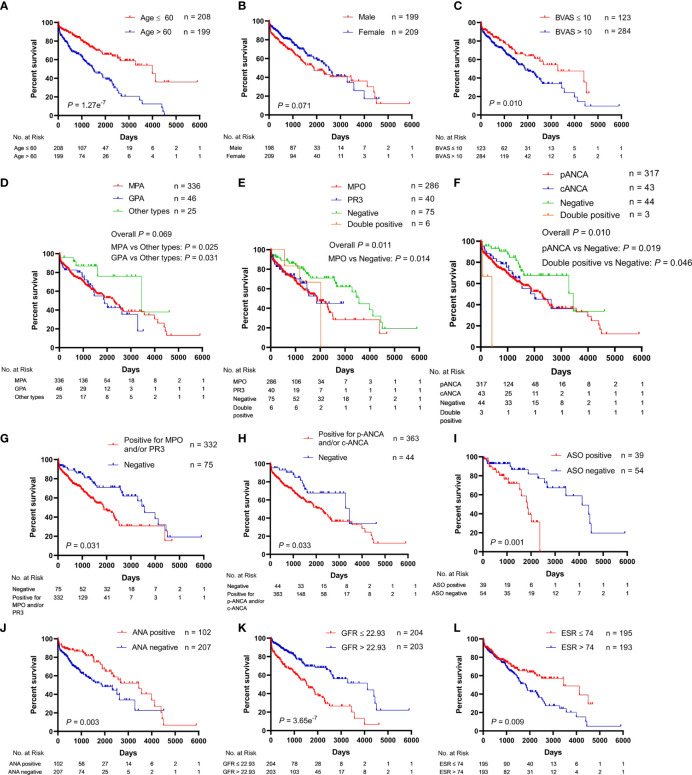
Association of clinical characteristics with overall survival in patients with antineutrophil cytoplasmic antibody (ANCA)-associated vasculitis. **(A)** Age. **(B)** Gender. **(C)** BVAS, Birmingham vasculitis activity score. **(D)** Pathological categories (MPA, GPA, and other types). MPA, microscopic polyangiitis; GPA, granulomatosis with polyangiitis. **(E)** Serum ANCA types (MPO, PR3, negative, and double positive for MPO and PR3) classified by the ELISA. MPO, myeloperoxidase; PR3, proteinase 3. **(F)** Serum ANCA types (p-ANCA, c-ANCA, negative, and double positive for p-ANCA and c-ANCA) classified by the indirect immunofluorescence (IIF) assay. p-ANCA, perinuclear ANCA; c-ANCA, cytoplasmic ANCA. **(G)** Negative *vs.* positive for MPO and/or PR3. **(H)** Negative *vs.* positive for p-ANCA and/or c-ANCA. **(I)** ASO, anti-streptolysin O. **(J)** ANA, antinuclear antibody. **(K)** GFR, glomerular filtration rate. **(L)** ESR, erythrocyte sedimentation rate.

**Figure 2:**
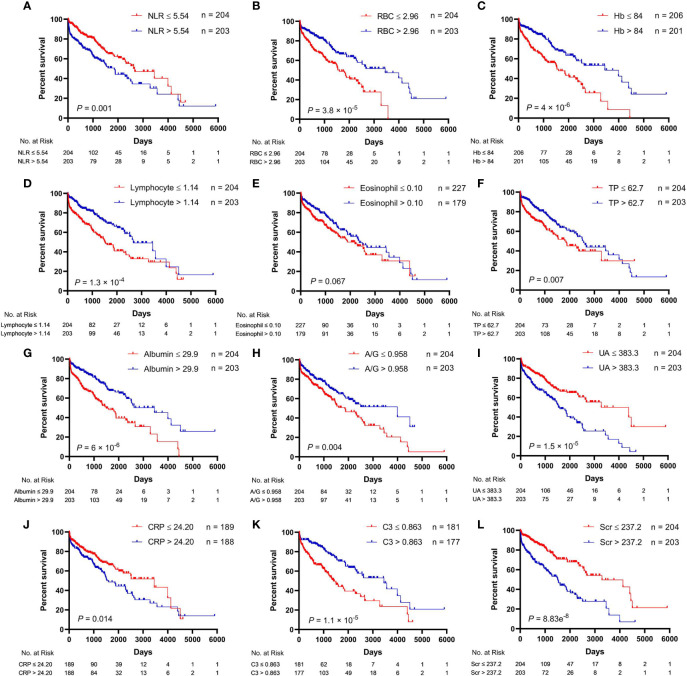
Relationships between hematological parameters and overall survival in patients with antineutrophil cytoplasmic antibody (ANCA)-associated vasculitis. **(A)** NLR, neutrophil-to-lymphocyte ratio. **(B)** RBC, red blood cell. **(C)** Hb, hemoglobin. **(D)** Lymphocyte. **(E)** Eosinophil. **(F)** TP, total protein. **(G)** Albumin. **(H)** A/G, albumin-to-globulin ratio. **(I)** UA, uric acid. **(J)** CRP, C-reactive protein. **(K)** C3, complement 3. **(L)** Scr, serum creatinine.

The univariate analysis for the association of clinical parameters with OS in AAV patients is shown in **Table S2**, and the Kaplan–Meier survival curves are shown in **Figures 1**, **2**. The clinical parameters with continuous variables except for BVAS were cut off by corresponding median values, including Scr, GFR, NLR, red blood cell (RBC), Hb, lymphocyte, eosinophil, total protein (TP), albumin, A/G, uric acid (UA), CRP, ESR, and C3. The mean (SE) and median (95% CI) OS for all the patients were 2,285 (172) and 2,290 (1,866–2,714) days, respectively. There was no significant difference in OS between MPA and GPA (*p* = 0.78). For the serum ANCA types classified by ELISA, negative MPO-ANCA and PR3-ANCA types indicated better OS as compared to MPO-ANCA alone (*p* = 0.014), as well as positive MPO and/or PR3 type (*p* = 0.031), and there was no significant difference in OS between MPO-ANCA alone and PR3-ANCA alone (*p* = 0.638). With the patterns observed by a means of IIF, negative p-ANCA and c-ANCA types predicted a favorable prognosis, and no significant difference was observed between p-ANCA alone and c-ANCA alone (*p* = 0.771). Positive ANA type was correlated with increased OS, while positive ASO type was correlated with decreased OS. BVAS > 10 served as a poor prognostic factor. Several clinical parameters were negatively correlated with OS, including Scr, NLR, UA, CRP, and ESR. The clinical parameters that were positively correlated with OS involved GFR, RBC, Hb, lymphocyte, TP, albumin, A/G, and C3. In addition, female AAV patients had marginally longer OS than male AAV patients (*p* = 0.071), and increased eosinophils were marginally associated with better OS (*p* = 0.067).

### Three-Year and Five-Year Survival Rates

For all of the AAV patients, the 1-year, 3-year, 5-year, and 10-year cumulative survival rates were 84.2%, 76.3%, 57.2%, and 32.4%, respectively. As shown in **Table S3**, the influence of the clinical parameters on OS was similar to that on 3-year survival rate as well as 5-year survival rate. There were no significant differences in the 3-year survival rate (*p* = 0.076) as well as 5-year survival rate (*p* = 0.119) among the pathological categories of AAV (MPA, GPA, and other types), whereas MPA showed a significantly decreased 3-year survival rate (*p* = 0.027) and marginally decreased 5-year survival rate (*p* = 0.0505) as compared to the other types. Moreover, GPA indicated a significantly decreased 5-year survival rate (*p* = 0.0498). In the serum ANCA types (ELISA), negative MPO-ANCA and PR3-ANCA types showed a higher 5-year survival rate as compared to MPO-ANCA alone (*p* = 0.005), PR3-ANCA alone (*p* = 0.007), and positive MPO-ANCA and/or PR3-ANCA type (*p* = 0.007), and there was a significant difference in the 5-year survival rate among the serum ANCA types (*p* = 0.008). In addition, negative MPO-ANCA and PR3-ANCA types indicated a higher 3-year survival rate as compared to MPO-ANCA alone (*p* = 0.005). No significant differences were observed in the 3-year survival rate (*p* = 0.0504) or 5-year survival rate (*p* = 0.243) among the ANCA IIF staining patterns consisting of p-ANCA, c-ANCA, and negative type. Positive ANA type was associated with a higher 3-year survival rate and 5-year survival rate. Conversely, positive ASO type was associated with a lower 3-year survival rate and 5-year survival rate. The 3-year and 5-year survival rates negatively correlated with age, BVAS, Scr, NLR, UA, and CRP. ESR only had a negative correlation with the 5-year survival rate. The other clinical parameters were positively correlated with 3-year and 5-year survival rates, including GFR RBC, Hb, lymphocyte, TP, albumin, and A/G. Compared to male AAV patients, female AAV patients had a marginally increased 5-year survival rate (*p* = 0.067). Additionally, increased eosinophils were associated with an elevated 3-year survival rate.

### Receiver Operating Characteristic Curves

**Figure 3:**
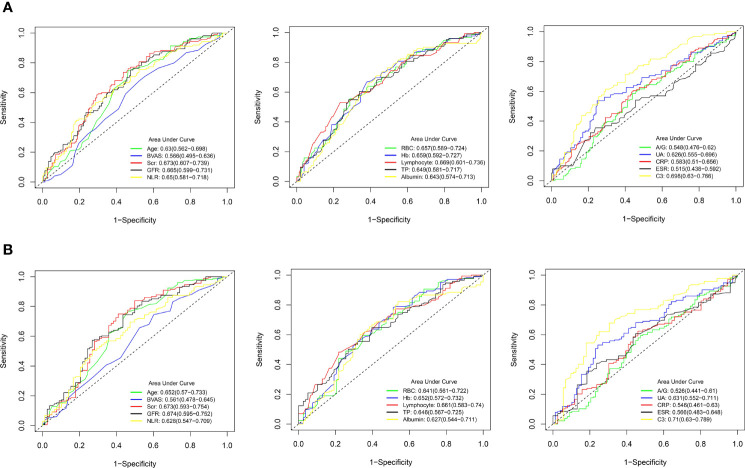
Receiver operating characteristic (ROC) curves for the predictive ability of clinical parameters with continuous variables in AAV patients. **(A)** 3-year survival rate. **(B)** 5-year survival rate. AAV, antineutrophil cytoplasmic antibody (ANCA)-associated vasculitis; BVAS, Birmingham vasculitis activity score; Scr, serum creatinine; GFR, glomerular filtration rate; NLR, neutrophil-to-lymphocyte ratio; RBC, red blood cell; Hb, hemoglobin; TP, total protein; A/G, albumin-to-globulin ratio; UA, uric acid; CRP, C-reactive protein; ESR, erythrocyte sedimentation rate; C3, complement 3.

ROC curves were plotted to evaluate the sensitivity, specificity, and accuracy of the clinical parameters with continuous variables in the prediction of 3-year and 5-year survival rates, including age, BVAS, Scr, GFR, NLR, RBC, Hb, lymphocyte, TP, albumin, A/G, UA, CRP, ESR, and C3 (**Figure 3**). The ROC curves were plotted using a time-independent model, and the area under the curve (AUC) was used to evaluate the predictive ability. For the ROC curves for the 3-year survival rate, the AUCs for BVAS, A/G, CRP, and ESR were all <0.6, implying that these clinical parameters had poor predictive value for the 3-year survival rate. In the ROC curves for the 5-year survival rate, only the AUC for C3 exceeded 0.7, suggesting that C3 had the strongest predictive ability among the clinical parameters.

### Establishment of a Nomogram

**Figure 4:**
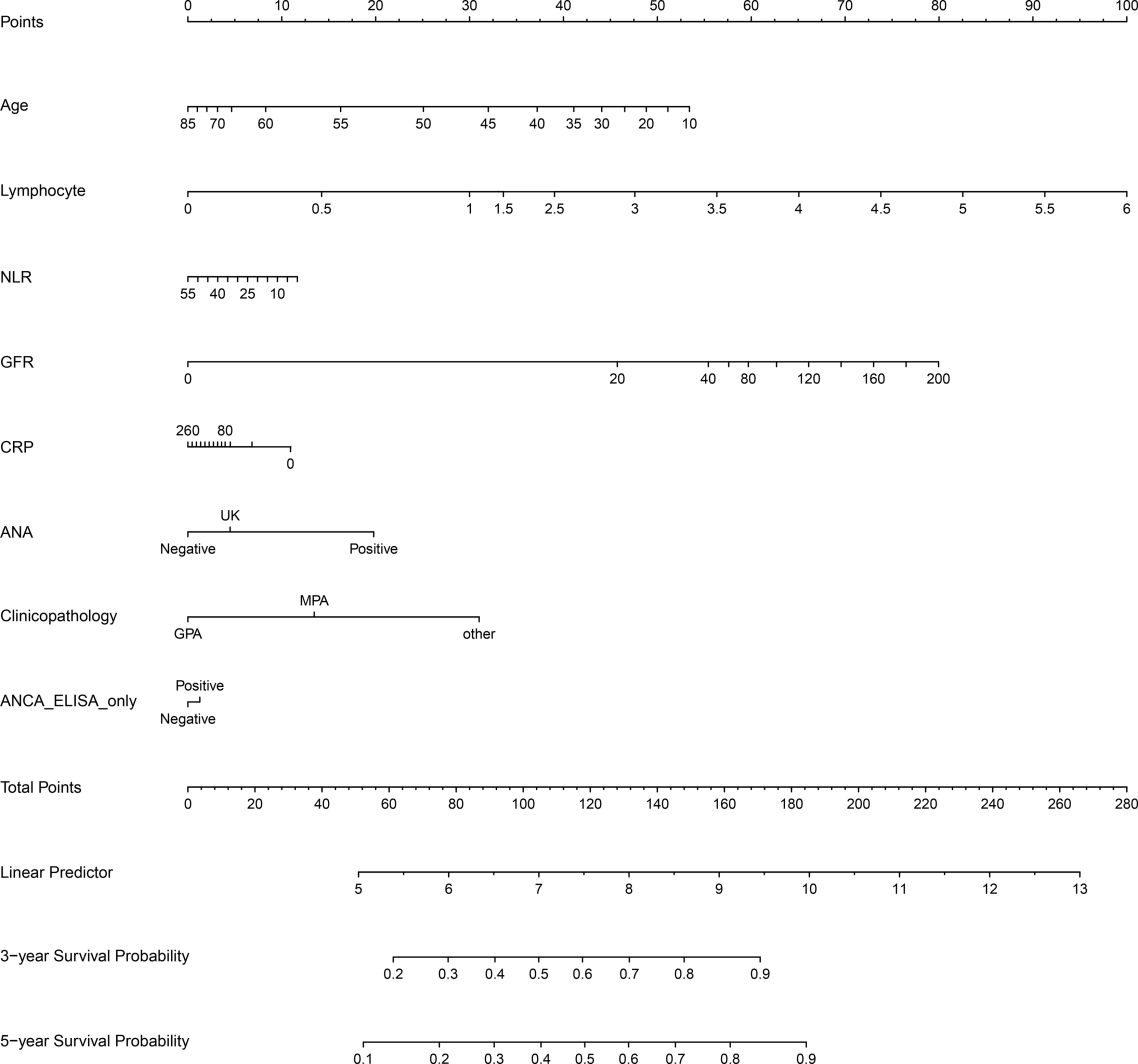
The nomogram for 3-year and 5-year survival rates in patients with antineutrophil cytoplasmic antibody (ANCA)-associated vasculitis. NLR, neutrophil-to-lymphocyte ratio; GFR, glomerular filtration rate; CRP, C-reactive protein; ASO, anti-streptolysin O; ANA, antinuclear antibody; ANCA ELISA only, serum ANCA types (negative or positive for MPO and/or PR3) only classified using ELISA. MPO, myeloperoxidase; PR3, proteinase 3; UK, unknown, meaning that a clinical parameter was assigned to a score corresponding to “UK” when the clinical parameter was not detected.

In order to construct a nomogram to predict 3-year and 5-year survival rates in AAV patients, independent prognostic factors were determined using multivariate Cox regression analysis. In summary of the above results, the clinical parameters influencing AAV prognosis included age, pathological categories (MPA, GPA, and other types), serum ANCA types (negative or positive for MPO-ANCA and/or PR3-ANCA), ANCA IIF staining patterns (negative or positive for p-ANCA and/or c-ANCA), BVAS, Scr, GFR, NLR, RBC, Hb, lymphocyte, TP, albumin, A/G, UA, CRP, ESR, and C3. As shown in **Table 2**, age, pathological categories, serum ANCA types (negative or positive for MPO-ANCA and/or PR3-ANCA), ANA, ASO, GFR, lymphocyte, NLR, and CRP were significant independent prognostic factors, and these clinical parameters except for ASO were used to construct the nomogram (**Figure 4**), in view of the fact that there were excessive missing values for ASO. In addition, the missing values in ANA were replaced with UK (unknown), and the missing values in CRP were replaced with the median value (24.20 mg/L). Each clinical parameter (negative, positive, or a value) reflected a designated score through an upward vertical line, and the point of intersection with the top “Points” line generated a score. All of the scores are added together to obtain a total score, which is present on the “Total Points” line. The 3-year and 5-year survival probabilities were obtained from the total score using a downward vertical line. The C-index (95% CI) of this nomogram was 0.721 (0.676–0.766), indicating high discrimination and a good predictive value of the nomogram.

**Table 2:** Multivariate Cox regression analysis for the association of clinical parameters with overall survival in AAV patients.

Clinical parameters	β	SE	Wald χ^2^	*p*-Value	HR	95% CI	
						Lower limit	Upper limit
Age	0.267	0.080	11.206	0.001	1.306	1.117	1.527
Clinicopathology							
Other types					1 (reference)		
MPA	0.282	1.902	0.022	0.882	1.326	0.032	55.152
GPA	8.121	3.001	7.325	0.007	3,365	9.393	1,205,515
ANCA ELISA only							
Negative					1 (reference)		
Positive for MPO and/or PR3	−3.498	0.941	13.824	0.0002	33.060	5.229	209.0188
ANA							
Negative					1 (reference)		
Positive	−4.305	1.266	11.569	0.001	0.013	0.001	0.161
ASO							
Negative							
Positive	4.490	1.981	5.134	0.023	89.077	1.833	4,327.722
GFR	−0.079	0.036	4.861	0.027	0.924	0.861	0.991
Lymphocyte	−2.203	0.879	6.286	0.012	0.110	0.020	0.618
NLR	0.284	0.146	3.812	0.048	1.329	0.999	1.768
UA	0.007	0.004	3.613	0.057	1.007	1.000	1.015
CRP	−0.064	0.019	11.403	0.001	0.938	0.904	0.974

AAV, antineutrophil cytoplasmic antibody (ANCA)-associated vasculitis; HR, hazard ratio; MPA, microscopic polyangiitis; GPA, granulomatosis with polyangiitis; MPO, myeloperoxidase; PR3, proteinase 3; IIF, indirect immunofluorescence; c-ANCA, cytoplasm-ANCA; p-ANCA, peripheral-ANCA; ANA, antinuclear antibody; ASO, anti-streptolysin O; GFR, glomerular filtration rate; NLR, neutrophil-to-lymphocyte ratio; UA, uric acid; CRP, C-reactive protein.

### Predictive Model Evaluation

**Figure 5:**
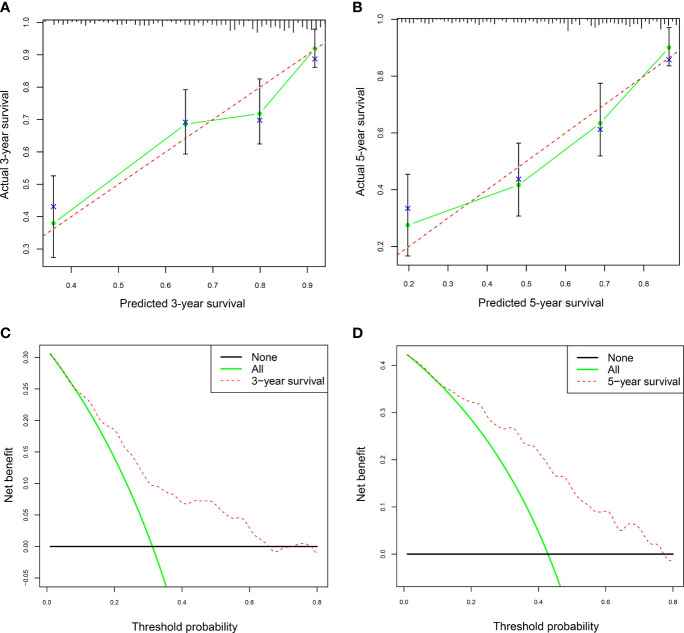
The calibration curves and decision curve analysis (DAC) of the nomogram in AAV. **(A)** The nomogram calibration curve for the 3-year survival rate in AAV patients. **(B)** The nomogram calibration curve for the 5-year survival rate in AAV patients. **(C)** Nomogram decision curve analysis (DCA) for the 3-year survival rate in AAV patients. **(D)** Nomogram DCA for the 5-year survival rate in AAV patients. AAV, antineutrophil cytoplasmic antibody (ANCA)-associated vasculitis. In panels **(A, B)**, the dashed lines serve as the reference lines. In panels **(C, D)**, the horizontal black line and green oblique line refer to no AAV patient and all AAV patients, respectively.

The calibration curves and DCA of the nomogram were used to evaluate the predictive model. The calibration curve analysis was performed through bootstrap sampling 1,000 times. The calibration curves for 3-year and 5-year survival rates had no significant deviation from the corresponding reference lines, indicating that the predicted values by the nomogram for 3-year and 5-year survival rates of AAV patients were generally consistent with the actual observed values (**Figures 5A, B**). The bias-corrected C-index was 0.684. DCA for 3-year and 5-year survival rates showed that high overall net benefits were observed within a wide range of thresholds (**Figures 5C, D**), indicating that the nomogram had good accuracy and applicability in the prediction of 3-year and 5-year survival rates for AAV patients.

## Discussion

As far as we know, the present study was the first to find that negative p-ANCA and c-ANCA types, RBC, TP, A/G, UA, ANA, and ASO were related to long-term survival in AAV. In particular with ASO, this study revealed that ASO was an independent prognostic factor and that positive ASO type was associated with shorter OS as well as lower 3-year and 5-year survival rates. In fact, the published studies on ASO were not many. The ASO antibody increases when the body frequently encounters beta-hemolytic streptococci and is easily quantified by a laboratory test (25). It has been reported that glomerulonephritis, tonsillitis/pharyngitis, and rheumatic fever, rather than streptococcus infection diseases, were leading diseases related to increased ASO titers (26). Behcet’s disease (BD) is a systemic immune disease and also a form of vasculitis. It was found that BD patients with high ASO titers were more likely to undergo tonsillitis and erythema nodosum (EN)-like lesions compared to BD patients with normal ASO titers (27). Moreover, the ASO test is a crucial micromethod for the serodiagnosis of rheumatic fever (28, 29), and patients with recurrent tonsillitis episodes and high ASO titers have a high prevalence of rheumatic heart disease (30). This suggests ASO plays a crucial role in rheumatic diseases. In our hospitals, ASO titers of 0~200 IU/ml were deemed as normal levels, namely, negative. For the current study, we illustrated that ASO had high predictive value for long-term prognosis in AAV. Regrettably, only 93 (22.9%) patients underwent ASO tests, as the ASO test is not an essential test in several hospitals.

ANA has been used for a central diagnostic principle of systemic lupus erythematosus (SLE) since 1975 and was first included as an important disease marker in the 1982 revised criteria for SLE, owing to ANA-induced renal damage through various mechanisms including receptor binding, cytokine stimulation, and immune complex deposition (31–36). It was reported that ANA was common in AAV and occurred in 19% of GPA patients, 47% of MPA patients, and 44% of AAV patients without renal involvement (37). Recently, Zhao et al. (21) found that AAV patients with positive ANA type had severer kidney impairment as well as worse renal clinical manifestations and pathological characteristics as compared with AAV patients with negative ANA type. In this study, ANA was first identified to be an independent predictive factor for long-term survival in AAV, indicating that the ANA test is useful and promising in AAV patients. In the nomogram, the missing values in ANA were replaced with the UK, meaning that a score was estimated through the “UK” point when the ANA test was not performed.

In the study, increased NLR was associated with a worse prognosis, while increased lymphocytes were associated with a better prognosis. Recently, Wacrenier et al. (38) demonstrated that lymphopenia was correlated with lower GFR, higher probability of kidney replacement therapy, severer glomerular lesions, and renal survival. It has been reported that higher NLR was correlated with a worse renal prognosis (17). Moreover, we illustrated that increasing GFR was related to better long-term prognosis while increasing Scr was related to worse long-term prognosis in AAV. Taken together, these implied a potential positive correlation between renal prognosis and long-term survival. With respect to RBC and Hb, previous studies found that lower Hb was associated with worse long-term survival (9, 39), and Hb is derived from erythrocytes and is primarily responsible for the transportation of oxygen and carbon dioxide (40, 41). In fact, RBC had a strong positive correlation with Hb in this study (r = 0.914, *p* = 9.05e^−161^). Both RBC and Hb were positively correlated with OS as well as 3-year and 5-year survival rates, indicating that anemia is a risk prognostic factor in AAV.

TP consisted of albumin and globulin, and A/G was the ratio of albumin to globulin. TP, albumin, and A/G were all associated with better prognosis, whereas globulin had no association with prognosis; it seemed that albumin played a core prognostic role. In fact, TP was positively correlated with albumin (r = 0.532, *p* = 3.74e^−31^), and A/G was strongly correlated with albumin (r = 0.708, *p* = 4.78e^−63^). With respect to the role of albumin in AAV, it has been reported that nephrotic hypoalbuminemia was associated with elevated all-cause mortality and reduced OS in AAV (42), and this finding was consistent with our result.

There were some limitations in the present study. For instance, the study lacks a validation cohort to validate the predictive ability of the nomogram; whether the prediction model is applicable to other populations is obscure. On the other hand, the nomogram contained several clinical parameters, including age, pathological categories, serum ANCA types (negative or positive for MPO and/or PR3), ANA, GFR, lymphocyte, NLR, and CRP. The prediction model is hard to be applied to AAV patients when some clinical parameters are not detected in a hospital. With regard to the clinical parameters used to construct the nomogram, age was negatively correlated with GFR (r = −0.234, *p* = 2 × 10^−6^), and NLR was negatively correlated with lymphocytes (r = −0.716, *p* = 2.77e^−65^), in spite of the fact that these clinical parameters were all significant independent prognostic factors.

## Conclusions

In summary, the clinical parameters influencing the long-term prognosis in AAV patients included age, pathological categories (MPA, GPA, and other types), ANCA IIF staining patterns (negative or positive for p-ANCA and/or c-ANCA), serum ANCA types (negative or positive for MPO and/or PR3), BVAS, Scr, GFR, NLR, RBC, Hb, lymphocyte, TP, albumin, A/G, UA, CRP, ESR, and C3. Among these clinical parameters, age, pathological categories, serum ANCA types, ANA, ASO, GFR, lymphocyte, NLR, and CRP were independent prognostic factors, and these clinical parameters except for ASO were used to establish a nomogram. The nomogram has a high predictive value for 3-year and 5-year survival rates. Our study revealed that laboratory examinations at diagnosis had great significance for predicting long-term prognosis in AAV patients.

## Data Availability Statement

The deidentified clinical data of AAV patients used and/or analyzed during the current study are available from the corresponding author upon reasonable request.

## Ethics Statement

The studies involving human participants were reviewed and approved by the independent Medical Ethics Committee of the People’s Hospital of Guilin (No. 2021-073KY) and the independent Medical Ethics Committee of the Affiliated Hospital of Guilin Medical University (No. 2022QTLL-05). Written informed consent from the participants’ legal guardian/next of kin was not required to participate in this study in accordance with the national legislation and the institutional requirements.

## Author Contributions

All authors contributed to the study’s conception and design. Q-QL, Y-FR, DQ, Y-JM, SC, JW, C-YW, X-JC, H-ZX, L-ZG, Y-YZ, H-XS, WZ, ZY, Y-FT, Z-JL, Z-NX, L-ML, H-JW, M-MZ, and F-NW were responsible for clinical data collection and follow-up. Among them, Q-QL and Y-FR were key contributors. K-WZ and PC were responsible for statistical analysis. The first draft of the manuscript was written by K-WZ, Y-HS was responsible for the modification and submission, and the other co-authors revised it critically for important intellectual content. All authors reviewed the manuscript and agreed on the final version of the article to be published. All authors agreed to be accountable for all aspects of the work in ensuring that questions related to the accuracy or integrity of any part of the work are appropriately investigated and resolved.

## Conflict of Interest

Author K-WZ was employed by Guangzhou Baiyunshan Pharmaceutical Holding Co., Ltd., Baiyunshan Pharmaceutical General Factory, Guangzhou, China. Author WZ was employed by Yangquan Coal Industry (Group) General Hospital, Yangquan, China.

The remaining authors declare that the research was conducted in the absence of any commercial or financial relationships that could be construed as a potential conflict of interest.

## Publisher’s Note

All claims expressed in this article are solely those of the authors and do not necessarily represent those of their affiliated organizations, or those of the publisher, the editors and the reviewers. Any product that may be evaluated in this article, or claim that may be made by its manufacturer, is not guaranteed or endorsed by the publisher.

## Abbreviations

ANCA, antineutrophil cytoplasmic antibody; AAV, ANCA-associated vasculitis; MPA, microscopic polyangiitis; GPA, granulomatosis with polyangiitis; MPO, myeloperoxidase; PR3, proteinase 3; IIF, indirect immunofluorescence; ANA, antinuclear antibody; ENA, extractable nuclear antigen; ASO, anti-streptolysin O; GFR, glomerular filtration rate; ESR, erythrocyte sedimentation rate; CRP, C-reactive protein; EMR, electronic medical record; OS, overall survival; NLR, neutrophil-to-lymphocyte ratio; EGPA, eosinophilic granulomatosis with polyangiitis; BVAS, Birmingham vasculitis activity score; WBC, white blood cell; C3, complement 3; Scr, serum creatinine; PLR, platelet-to-lymphocyte ratio; CTX, cyclophosphamide; MLR, monocyte-to-lymphocyte ratio; A/G, albumin-to-globulin ratio; ROC, receiver operating characteristic; RBC, red blood cell; Hb, hemoglobin; TP, total protein; UA, uric acid; p-ANCA, perinuclear ANCA; c-ANCA, cytoplasmic ANCA; AUC, area under the curve; C-index, index of concordance; BD, Behcet’s disease; EN, erythema nodosum; SLE, systemic lupus erythematosus; ALT, alanine aminotransferase; AST, aspartate aminotransferase; TBIL, total bilirubin; DBIL, direct bilirubin; IBIL, indirect bilirubin; BUN, blood urea nitrogen; C4, complement 4; IgA, immunoglobulin A; IgG, immunoglobulin G; IgM, immunoglobulin M; RF, rheumatoid factor.
