# Supplementary material for: Long-Term Prognostic Factors in Patients With Antineutrophil Cytoplasmic Antibody-Associated Vasculitis: A 15-Year Multicenter Retrospective Study
Source: Front Immunol. 2022 Jun 30;13:913667. doi: 10.3389/fimmu.2022.913667 (PMC9279612; doi:10.3389/fimmu.2022.913667)
Supplement: Supplementary file 1 [file Table_1.pdf]

Table S1. Relationships between pathological categories (MPA, GPA, and other types) and serum ANCA types (MPO, PR3, negative, and double positive for MPO and PR3) in AAV patients.

	MPO	PR3	Double positive	Negative	In total
MPA	275 (81.8%)	15 (4.5%)	2 (0.6%)	44 (13.1%)	336 (100.0%)
GPA	8 (17.4%)	22 (47.8%)	4 (8.7%)	12 (26.1%)	46 (100.0%)
Other types	3 (12.0%)	3 (12.0%)	0 (0.00%)	19 (76.0%)	25 (100.0%)

Abbreviations: AAV, antineutrophil cytoplasmic antibody (ANCA)-associated vasculitis; MPA, microscopic polyangiitis; GPA, granulomatosis with polyangiitis; MPO, myeloperoxidase; PR3, proteinase 3.

Note: Each percentage represents the proportion of patients in AAV patients with the corresponding pathological category. For instance, 81.8% MPA patients tested positive for MPO-ANCA, and 47.8% GPA patients tested positive for PR3-ANCA.
