# Supplementary material for: Long-Term Prognostic Factors in Patients With Antineutrophil Cytoplasmic Antibody-Associated Vasculitis: A 15-Year Multicenter Retrospective Study
Source: Front Immunol. 2022 Jun 30;13:913667. doi: 10.3389/fimmu.2022.913667 (PMC9279612; doi:10.3389/fimmu.2022.913667)
Supplement: Supplementary file 2 [file Table_2.pdf]

Table S2. Cox regression analysis for influences of clinical parameters on the overall survival in AAV patients.

Clinical parameters	N	Mean ± SE (days)	Median (days, 95% CI)	HR (95% CI)	P value
Age, years					
≤ 60	208	3479 ± 288	3993 (2706 – 5280)	1 (reference)	
> 60	199	1758 ± 150	1496 (1040 – 1951)	2.453 (1.758 – 3.422)	$1.27e^{-7}$
Gender					
Male	199	2469 ± 222	1890 (1239 – 2541)	1 (reference)	
Female	209	2450 ± 177	2500 (2092 – 2908)	0.745 (0.541 – 1.026)	0.071
BVAS					
≤ 10	123	2895 ± 215	3282 (1830 – 4734)	1 (reference)	
> 10	284	2275 ± 194	1885 (1368 – 2402)	1.616 (1.120 – 2.331)	0.010
Clinicopathology					
Other types	25	3297 ± 460	3450 (829 – 6071)	1 (reference)	

MPA	336	1975 ± 213	1893 (1269 – 2517)	2.701 (1.103 – 6.613)	0.025
GPA	46	2538 ± 188	2290 (1805 – 2775)	2.885 (1.090 – 7.634)	0.031
ANCA ELISA only					
Negative	75	3288 ± 294	3561 (3109 – 4013)	1 (reference)	
MPO	286	2158 ± 174	1890 (1342 – 2438)	1.759 (1.120 – 2.761)	0.014
PR3	40	1806 ± 215	1855 (NA)	0.919 (0.964 – 1.943)	0.964
Double positive	6	1632 ± 286	2017 (NA)	2.045 (0.479 – 8.731)	0.334
Negative	75	3339 ± 398	3561 (1604 – 5517)	1 (reference)	
Positive for MPO and/or PR3	332	2259 ± 138	3561 (1339 – 2447)	1.635 (1.046 – 2.556)	0.031
ANCA IIF only					
Negative	44	3035 ± 321	3450 (1587 – 5313)	1 (reference)	
p-ANCA	317	2485 ± 191	2284 (1887 – 2681)	2.090 (1.128 – 3.872)	0.019

c-ANCA	43	2034 ± 227	2017 (899 - 3135)	1.098 (0.484 – 2.490)	0.823
Double positive	3	301 ± 132	416 (NA)	4.664 (1.028 – 21.161)	0.046
Negative	44	3231 ± 326	NA	1 (reference)	
Positive for p-ANCA and/or c-ANCA	363	2438 ± 170	2284 (1827 – 2741)	1.952 (1.057 – 3.608)	0.033
ANA					
Negative	207	2107 ± 208	1893 (1089 – 2697)	1 (reference)	
Positive	102	2944 ± 245	3450 (2301 – 4599)	0.529 (0.348 – 0.806)	0.003
ASO					
Negative	54	3696 ± 343	3450 (2301 – 4599)	1 (reference)	
Positive	39	1621 ± 144	1893 (1089 – 2697)	2.101 (1.343 – 3.268)	0.001
Scr, μmol/L					
≤ 237.2	204	3260 ± 247	3282 (1807 – 4757)	1 (reference)	

> 237.2	203	1784 ± 153	1507 (1192 – 1822)	2.479 (1.777 – 3.457)	$8.83e^{-8}$
GFR, mL/min					
≤ 22.93	204	1795 ± 149	1529 (1091 – 1967)	1 (reference)	
> 22.93	203	3292 ± 252	4126 (1955 – 6297)	0.421 (0.301 – 0.587)	$3.65e^{-7}$
NLR					
≤ 5.54	204	2699 ± 177	2623 (1740 – 3506)	1 (reference)	
> 5.54	203	2251 ± 230	1855 (1507 – 2203)	1.705 (1.235 – 2.355)	0.001
RBC, $\times10^{12}$/L					
≤ 2.96	204	1742 ± 124	1599 (1294 – 1904)	1 (reference)	
> 2.96	203	3117 ± 235	3450 (2055 – 4845)	0.495 (0.354 – 0.692)	3.8×10^{-5}
Hb, g/L					
≤ 84	206	1796 ± 155	1538 (1002 – 2073)	1 (reference)	
> 84	201	3202 ± 249	3450 (1925 – 4975)	0.458 (0.329 – 0.639)	4×10^{-6}

Lymphocyte, $\times 10^9$/L					
≤ 1.14	204	2073 ± 167	1507 (1105 – 1909)	1 (reference)	
> 1.14	203	2929 ± 272	2667 (1831 – 3503)	0.528 (0.381 – 0.733)	1.3×10^{-4}
Eosinophil, $\times 10^9$/L					
≤ 0.10	227	2267 ± 177	1885 (1173 – 2597)	1 (reference)	
> 0.10	179	2696 ± 227	2367 (1719 – 3015)	0.738 (0.534 – 1.021)	0.067
TP, g/L					
≤ 62.7	204	2230 ± 203	1855 (1240 – 2470)	1 (reference)	
> 62.7	203	2792 ± 213	2565 (2222 – 2908)	0.640 (0.464 – 0.884)	0.007
Albumin, g/L					
≤ 29.9	204	1890 ± 167	1496 (1027 – 1965)	1 (reference)	
> 29.9	203	3177 ± 257	3450 (2304 – 4596)	0.468 (0.336 – 0.650)	6×10^{-6}
A/G					

≤ 0.958	204	2130 ± 185	1855 (1172 – 2538)	1 (reference)	
> 0.958	203	2850 ± 184	3993 (1651 – 6335)	0.619 (0.448 – 0.855)	0.004
UA, μmol/L					
≤ 383.3	204	3345 ± 275	3282 (1674 – 4890)	1 (reference)	
> 383.3	203	1868 ± 153	1538 (1217 – 1858)	2.059 (1.486 – 2.855)	1.5×10^{-5}
CRP, mg/L					
≤ 24.20	189	2685 ± 182	3450 (1865 – 5035)	1 (reference)	
> 24.20	188	2264 ± 219	1529 (1156 – 1902)	1.512 (1.086 – 2.104)	0.014
ESR, mm/h					
≤ 74	195	2890 ± 189	3450 (1878 – 5022)	1 (reference)	
> 74	193	2103 ± 184	1807 (1497 – 2117)	1.554 (1.115 – 2.167)	0.009
C3, g/L					
≤ 0.863	181	1952 ± 190	1400 (1078 – 1722)	1 (reference)	

> 0.863	177	3123 ± 179	3450 (2410 – 4490)	0.458 (0.323 – 0.649)	1.1×10^{-5}

Abbreviations: AAV, antineutrophil cytoplasmic antibody (ANCA)-associated vasculitis; SE, standard error; CI, confidence interval; HR, hazard ratio; BVAS, Birmingham vasculitis activity score; MPA, microscopic polyangiitis; GPA, granulomatosis with polyangiitis; ELISA, enzyme linked immunosorbent assay; MPO, myeloperoxidase; PR3, proteinase 3; IIF, indirect immunofluorescence; c-ANCA, cytoplasm-ANCA; p-ANCA, peripheral-ANCA; ANA, antinuclear antibody; ASO, anti-streptolysin O; Scr, serum creatinine; GFR, glomerular filtration rate; NLR, neutrophil-to-lymphocyte ratio; RBC, red blood count; Hb, hemoglobin; TP, total protein; A/G, albumin-to-globulin ratio; UA, uric acid; CRP, C-reactive protein; ESR, erythrocyte sedimentation rate; C3, complement 3.
