# Supplementary material for: Long-Term Prognostic Factors in Patients With Antineutrophil Cytoplasmic Antibody-Associated Vasculitis: A 15-Year Multicenter Retrospective Study
Source: Front Immunol. 2022 Jun 30;13:913667. doi: 10.3389/fimmu.2022.913667 (PMC9279612; doi:10.3389/fimmu.2022.913667)
Supplement: Supplementary file 3 [file Table_3.pdf]

**Table S3. Correlations of clinical parameters with 3-year and 5-year cumulative survival rates in AAV patients.**

| Clinical parameters | N | 3-year survival rate | RR (95% CI) | P value | 5-year survival rate | RR (95% CI) | P value |
|---|---|---|---|---|---|---|---|
| Age, years | | | | | | | |
| ≤ 60 | 208 | 78.1% | 1 (reference) | | 68.4% | 1 (reference) | |
| > 60 | 199 | 58.9% | 2.468 (1.602 – 3.803) | $3.4 \times 10^{-5}$ | 43.7% | 2.152 (1.418 – 3.263) | $2.8 \times 10^{-4}$ |
| Gender | | | | | | | |
| Male | 199 | 63.7% | 1 (reference) | | 51.7% | 1 (reference) | |
| Female | 209 | 70.9% | 0.727 (0.480 – 1.101) | 0.132 | 61.0% | 0.693 (0.468 – 1.026) | 0.067 |
| BVAS | | | | | | | |
| ≤ 10 | 123 | 76.5% | 1 (reference) | | 63.9% | 1 (reference) | |
| > 10 | 284 | 65.2% | 1.735 (1.071 – 2.810) | 0.024 | 50.7% | 1.746 (1.129 – 2.699) | 0.012 |
| Clinicopathology | | | ` | | | | |

| | | | | | | | |
|---|---|---|---|---|---|---|---|
| Other types | 25 | 86.8% | 1 (reference) | | 76.0% | 1 (reference) | |
| MPA | 336 | 66.8% | 3.667 (1.075 – 12.512) | 0.027 | 55.9% | 2.493 (0.971 – 6.399) | 0.0505 |
| GPA | 46 | 72.2% | 2.889 (0.737 – 11.328) | 0.117 | 51.1% | 2.903 (0.981 – 8.590) | 0.0498 |
| ANCA ELISA only | | | | | | | |
| Negative | 75 | 80.9% | 1 (reference) | | 71.0% | 1 (reference) | |
| MPO | 286 | 64.5% | 2.415 (1.287 – 4.532) | 0.005 | 52.9% | 2.154 (1.244 – 3.728) | 0.005 |
| PR3 | 40 | 70.0% | 1.867 (0.766 – 4.554) | 0.166 | 45.0% | 2.944 (1.327 – 6.533) | 0.007 |
| Double positive | 6 | 66.7% | 2.179 (0.362 – 13.101) | 0.338 | — | | |
| Negative | 75 | 76.4% | 1 (reference) | | 66.9% | 1 (reference) | |
| Positive for MPO and/or PR3 | 332 | 66.9% | 1.569 (0.881 – 2.794) | 0.124 | 53.6% | 1.730 (1.022 – 2.929) | 0.040 |
| ANCA IIF only | | | | | | | |
| Negative | 44 | 84.7% | 1 (reference) | | 67.6% | 1 (reference) | |

| | | | | | | | |
|---|---|---|---|---|---|---|---|
| p-ANCA | 317 | 66.3% | 2.693 (1.162 – 6.243) | 0.017 | 56.7% | 1.631 (0.833 – 3.194) | 0.151 |
| c-ANCA | 43 | 72.0% | 2.046 (0.718 – 5.831) | 0.176 | 50.3% | 2.045 (0.855 – 4.891) | 0.106 |
| Double positive | 3 | — | | | — | | |
| Negative | 44 | 84.7% | 1 (reference) | | 67.6% | 1 (reference) | |
| Positive for p-ANCA and/or c-ANCA | 363 | 66.7% | 2.643 (1.145 – 6.102) | 0.019 | 56.0% | 1.689 (0.867 – 3.292) | 0.121 |
| ANA | | | | | | | |
| Negative | 207 | 61.3% | 1 (reference) | | 50.3% | 1 (reference) | |
| Positive | 102 | 85.0% | 0.274 (0.148 – 0.506) | $1.8 \times 10^{-5}$ | 72.4% | 0.382 (0.229 – 0.638) | $1.9 \times 10^{-4}$ |
| ASO | | | | | | | |
| Negative | 54 | 89.5% | 1 (reference) | | 81.9% | 1 (reference) | |
| Positive | 39 | 71.8% | 3.143 (1.048 – 9.427) | 0.035 | 52.2% | 4.180 (1.649 – 10.599) | 0.002 |

| | | | | | | | |
|---|---|---|---|---|---|---|---|
| Scr, µmol/L | | | | | | | |
| ≤ 237.2 | 204 | 78.6% | 1 (reference) | | 69.2% | 1 (reference) | |
| > 237.2 | 203 | 58.6% | 2.567 (1.661 – 3.966) | $1.7 \times 10^{-5}$ | 42.5% | 2.260 (1.508 – 3.388) | $5.52e^{-8}$ |
| GFR, mL/min | | | | | | | |
| ≤ 22.93 | 204 | 60.4% | 1 (reference) | | 44.6% | 1 (reference) | |
| > 22.93 | 203 | 77.0% | 0.458 (0.298 – 0.703) | $3.2 \times 10^{-4}$ | 68.0% | 0.379 (0.253 – 0.568) | $2 \times 10^{-6}$ |
| NLR | | | | | | | |
| ≤ 5.54 | 204 | 77.2% | 1 (reference) | | 63.5% | 1 (reference) | |
| > 5.54 | 203 | 60.3% | 2.218 (1.442 – 3.410) | $2.5 \times 10^{-4}$ | 49.0% | 1.845 (1.241 – 2.743) | 0.002 |
| RBC, $\times 10^{12}$/L | | | | | | | |
| ≤ 2.96 | 204 | 59.9% | 1 (reference) | | 47.3% | 1 (reference) | |
| > 2.96 | 203 | 77.4% | 0.436 (0.283 – 0.671) | $1.4 \times 10^{-4}$ | 66.5% | 0.448 (0.300 – 0.668) | $7.5 \times 10^{-5}$ |
| Hb, g/L | | | | | | | |

| | | | | | | | |
|---|---|---|---|---|---|---|---|
| ≤ 84 | 206 | 58.0% | 1 (reference) | | 46.2% | 1 (reference) | |
| > 84 | 201 | 79.3% | 0.361 (0.233 – 0.560) | $4 \times 10^{-6}$ | 66.2% | 0.438 (0.293 – 0.653) | $4.6 \times 10^{-5}$ |
| Lymphocyte, $\times 10^9$/L | | | | | | | |
| ≤ 1.14 | 204 | 59.9% | 1 (reference) | | 45.6% | 1 (reference) | |
| > 1.14 | 203 | 77.8% | 0.424 (0.275 – 0.654) | $8.7 \times 10^{-5}$ | 67.5% | 0.404 (0.270 – 0.604) | $8 \times 10^{-6}$ |
| Eosinophil, $\times 10^9$/L | | | | | | | |
| ≤ 0.10 | 227 | 63.6% | 1 (reference) | | 52.8% | 1 (reference) | |
| > 0.10 | 179 | 74.9% | 0.583 (0.378 – 0.898) | 0.014 | 60.6% | 0.737 (.496 – 1.097) | 0.132 |
| TP, g/L | | | | | | | |
| ≤ 62.7 | 204 | 61.3% | 1 (reference) | | 50.1% | 1 (reference) | |
| > 62.7 | 203 | 75.9% | 0.503 (0.328 – 0.772) | 0.002 | 62.4% | 0.598 (0.403 – 0.888) | 0.011 |
| Albumin, g/L | | | | | | | |
| ≤ 29.9 | 204 | 58.9% | 1 (reference) | | 46.3% | 1 (reference) | |

| | | | | | | | |
|---|---|---|---|---|---|---|---|
| > 29.9 | 203 | 78.7% | 0.384 (0.248 – 0.594) | $1.3 \times 10^{-5}$ | 66.2% | 0.440 (0.295 – 0.657) | $5.1 \times 10^{-5}$ |
| A/G | | | | | | | |
| ≤ 0.958 | 204 | 63.8% | 1 (reference) | | 49.3% | 1 (reference) | |
| > 0.958 | 203 | 73.9% | 0.621 (0.406 – 0.948) | 0.027 | 63.3% | 0.575 (0.387 – 0.853) | 0.006 |
| UA, µmol/L | | | | | | | |
| ≤ 383.3 | 204 | 75.2% | 1 (reference) | | 65.1% | 1 (reference) | |
| > 383.3 | 203 | 61.9% | 1.833 (1.198 – 2.805) | 0.005 | 46.8% | 2.130 (1.429 – 3.173) | $1.85 \times 10^{-4}$ |
| CRP, mg/L | | | | | | | |
| ≤ 24.20 | 189 | 74.4% | 1 (reference) | | 64.5% | 1 (reference) | |
| > 24.20 | 188 | 63.3% | 1.703 (1.095 – 2.650) | 0.018 | 47.2% | 2.025 (1.340 – 3.062) | 0.001 |
| ESR, mm/h | | | | | | | |
| ≤ 74 | 195 | 72.7% | 1 (reference) | | 63.8% | 1 (reference) | |
| > 74 | 193 | 66.0% | 1.392 (0.903 – 2.148) | 0.134 | 49.7% | 1.765 (1.176 – 2.648) | 0.006 |

| | | | | | | | |
|---|---|---|---|---|---|---|---|
| C3, g/L | | | | | | | |
| ≤ 0.863 | 181 | 59.1% | 1 (reference) | | 41.8% | 1 (reference) | |
| > 0.863 | 177 | 79.6% | 0.369 (0.231 – 0.591) | $2.5 \times 10^{-5}$ | 68.9% | 0.326 (0.211 – 0.504) | $2.97e^{-7}$ |

**Abbreviations:** AAV, antineutrophil cytoplasmic antibody (ANCA)-associated vasculitis; SE, standard error; CI, confidence interval; RR, risk ratio; BVAS, Birmingham vasculitis activity score; MPA, microscopic polyangiitis; GPA, granulomatosis with polyangiitis; ELISA, enzyme linked immunosorbent assay; MPO, myeloperoxidase; PR3, proteinase 3; IIF, indirect immunofluorescence; c-ANCA, cytoplasm-ANCA; p-ANCA, peripheral-ANCA; ANA, antinuclear antibody; ASO, anti-streptolysin O; Scr, serum creatinine; GFR, glomerular filtration rate; NLR, neutrophil-to-lymphocyte ratio; RBC, red blood count; Hb, hemoglobin; TP, total protein; A/G, albumin-to-globulin ratio; UA, uric acid; CRP, C-reactive protein; ESR, erythrocyte sedimentation rate; C3, complement 3.
